# Quantified Self and Comprehensive Geriatric Assessment: Older Adults Are Able to Evaluate Their Own Health and Functional Status

**DOI:** 10.1371/journal.pone.0100636

**Published:** 2014-06-26

**Authors:** Olivier Beauchet, Cyrille P. Launay, Christine Merjagnan, Anastasiia Kabeshova, Cédric Annweiler

**Affiliations:** 1 Department of Neuroscience, Angers University Hospital, Angers, France; 2 Biomathics, Paris, France; 3 Department of Medical Biophysics, the University of Western Ontario, London, Canada; University Of São Paulo, Brazil

## Abstract

**Background:**

There is an increased interest of individuals in quantifying their own health and functional status. The aim of this study was to examine the concordance of answers to a self-administered questionnaire exploring health and functional status with information collected during a full clinical examination performed by a physician among cognitively healthy adults (CHI) and older patients with mild cognitive impairment (MCI) or mild-to-moderate Alzheimer disease (AD).

**Methods:**

Based on cross-sectional design, a total of 60 older adults (20 CHI, 20 patients with MCI, and 20 patients with mild-to-moderate AD) were recruited in the memory clinic of Angers, France. All participants completed a self-administered questionnaire in paper format composed of 33 items exploring age, gender, nutrition, place of living, social resources, drugs daily taken, memory complaint, mood and general feeling, fatigue, activities of daily living, physical activity and history of falls. Participants then underwent a full clinical examination by a physician exploring the same domains.

**Results:**

High concordance between the self-administered questionnaire and physician's clinical examination was showed. The few divergences were related to cognitive status, answers of AD and MCI patients to the self-administered questionnaire being less reliable than those of CHI.

**Conclusion:**

Older adults are able to evaluate their own health and functional status, regardless of their cognitive status. This result needs to be confirmed and opens new perspectives for the quantified self-trend and could be helpful in daily clinical practice of primary care.

## Introduction

Quantified self (QS) is a recent trend in general population based on self-measure of health and functional status using new digital technologies to become healthier or remain healthy [1]. Nowadays, the miniaturization of devices combined with new digital technologies allow the measure of human physiological parameters reflecting health status (e.g., caloric expenditures or blood pressure). The advantage of this “high-tech” QS is to provide objective measures, but its main disadvantage is to consider the individual more as a measurement object, than an actor of his own health, the latter point being yet crucial for health improvement. It has been reported that improvements of health and functional status, as well as reduced adverse consequences on health systems, depend in part on the active participation of individuals [2]–[4]. For this reason, the World Health Organization (WHO) recommends the use of self-administered questionnaires to rate and monitor individuals' own health [4]–[6]. This approach is also thought to educate people about wellness and promote healthy lifestyles [2]–[6]. Because of the increasing popularity of QS, self-administered questionnaires evaluating health and functional status in complement or not to high-tech QS could be an interesting solution to improve older adults' health and functional status, and thus to limit adverse consequences of age-related disorders on health systems. However, an obstacle to this self-rated health approach could be the decline of cognition encountered in older adults, which may affect their ability to provide answers objectively reflecting their actual situation.

Older adults' health and functional status is heterogeneous because of the various cumulative effects of chronic diseases and physiologic decline, contributing to a vicious cycle of increased frailty [7]–[9]. Thanks to advances in medicine and hygiene, a growing number of older adults spend more years with a greater range of disorders causing disability but not mortality [10]. Health systems thus need to face this new challenge [10], [11]. Quantification of the burden of non-fatal health outcomes is crucial to understand how efficiently health systems may respond to this situation.

In daily practice, adapted care plans for older patients arise from an assessment process called comprehensive geriatric assessment (CGA), which is a multidimensional, interdisciplinary diagnostic process to determine the medical, psychological, and functional capabilities of older adults [12], [13]. The implementation of systematic CGA among older community-dwellers with accumulation of chronic diseases and disability remains difficult in daily practice because of a number of issues. First, while the number of older community-dwellers keeps increasing, the number of health care professionals with geriatric skills does not [14], [15]. Second, the CGA is a complex and time-consuming process [15], [16]. Third, the CGA requires multidisciplinary geriatric teams that cannot support alone the care of all older adults due to their limited number [13]–[16].

Because of these issues preventing performing a CGA in every older community-dweller with cumulative chronic diseases, and because of the increased interest of individuals in their own health and functional status, we hypothesized that it was possible to perform a self-CGA using a self-administered questionnaire among older adults with and without cognitive decline. The aim of this study was to examine, among cognitively healthy adults (CHI) and older patients with mild cognitive impairment (MCI) or mild-to-moderate Alzheimer disease (AD), the concordance of answers to a self-administered questionnaire with information collected by a physician during a full clinical examination.

## Methods

### Participants

Between March and May 2013, 60 older adults (i.e., 20 CHI, 20 patients with MCI, and 20 patients with mild-to-moderate AD) were recruited in this cross-sectional study. All participants were sent for a memory complaint by their primary care physician to the memory clinic of Angers University Hospital, France. Eligibility criteria were age 65 years and over, outpatients, able to understand and speak French, and no acute medical illness in the past month. For the present analysis, exclusion criteria were severe AD (i.e., Mini-Mental State Examination score (MMSE) ≤ 9), low near vision, neurological diseases including Parkinson's disease, cerebellar disease, myelopathy, peripheral neuropathy, and major orthopaedic impairments of the upper limbs [17].

### Self-administered questionnaire

A self-administered questionnaire in format paper was given to each patient meeting the selection criteria at their arrival in the memory clinic. This questionnaire consisted of 33 items (Table S1). Except age and weight, all items corresponded to a question with a forced choice in closed-ended format (i.e., yes or no, or calling for a specific answer). The French version of questionnaire is presented in Appendix S1.

### Neuropsychological assessment

Neuropsychological assessment was performed during a face-to-face examination carried out by a neuropsychologist. The memory complaint was characterized using the same 6 questions exploring memory as for the self-administered questionnaire. All answers were coded as a binary variable (i.e., yes or no). In addition, the following standardized tests were used to probe several aspects of cognition: MMSE, frontal assessment battery (FAB), ADAS-cog, trail making test parts A and B and French version of the free and cued selective reminding test [17]–[22]. The diagnoses of MCI and AD were made during multidisciplinary meetings involving geriatricians, neurologists and neuropsychologists of Angers University Memory Clinic, and were based on the neuropsychological tests mentioned above, medical examination findings, blood tests and Magnetic Resonance Imaging (MRI) of the brain. MCI was diagnosed according to Winblad et al. consensus criteria [23]. Participants with all categories of MCI were included in this study, i.e. amnestic and non-amnestic as well as single and multiple affected domains. The diagnosis of AD followed the DSM-IV and NINCDS/ADRDA consensus criteria [24]. Mild and moderate stages of AD were defined as MMSE score ≥ 10. Participants who were neither MCI nor dementia/AD and who had normal neuropsychological and functional performance were considered as CHI, regardless of the presence or not of underlying non-cognitive chronic diseases.

### Medical examination

Participants underwent a full clinical examination by a physician. Age, gender, weight (kg), height (m), the number and the type of therapeutic classes of drugs used per day were recorded. The body mass index (BMI, in kg/m^2^) was calculated based on anthropometric measurements. A loss of 4 kg and over in past year was also sought. The usual place of life (i.e., home-living versus institution-dwelling defined as living in nursing homes or in senior housing facilities), and the use of formal and/or informal home and social services were also recorded and coded as a binary variable (i.e., yes or no). Activities of daily living scale (ADL) and instrumental activities of daily living scale (IADL) were performed [25], [26]. Depression was evaluated by the 4-item short Geriatric Depression Scale (GDS) score [27]. A score ≥1 indicated symptoms of depression. Participants were also questioned on their feeling and fatigue using the same questions as for the self-administered questionnaire. Physical activity was considered if participants practiced at least one recreational physical (walking, gymnastics, cycling, swimming or gardening) activity for at least one hour a week for the past month or more. Participants were also interviewed on their history of falls over the past year. A fall was defined as an event resulting in a person coming to rest unintentionally on the ground or at other lower level, not as the result of a major intrinsic event or an overwhelming hazard, according to the French society of geriatrics and gerontology (SFGG) and the French national agency for health (HAS) [28]. In the case of falls, the severity was recorded using the same items as for the self-administered questionnaire. In addition, education level was evaluated with the number of years of school completed.

### Standard Protocol Approvals, Registrations, and Participant Consents

In the present study, a written informed consent was obtained from the patients themselves in the presence of their trusted person, usually a family member, who helped them to make decision. The study was conducted in accordance with the ethical standards set forth in the Helsinki Declaration (1983). The entire study protocol was approved by the Ethical Committee of Angers Hospital University, France (2013/25 – “Auto-évaluation de l'état de santé de la personne âgée”).

### Statistics

Participants' baseline characteristics were summarized using means and standard deviations or frequencies and percentages, as appropriate. Participants were separated into three groups based on their cognitive status (i.e., CHI, MCI and AD). A second stratification into two groups of all participants based on their education level and using a threshold of the median value (i.e., 11 years spent at school) was also done. Comparisons between groups of participants were performed using Kruskal-Wallis or one-way analysis of variance with Bonferroni corrections, *t*-test, Mann-Whitney test, and Chi-Square test, as appropriate. Comparison of answers from the self-administered questionnaire and the full clinical examination were performed using paired *t*-tests, Wilcoxon signed rank test or McNemar test, as appropriate.

## Results

Table S2 reports the characteristics of participants obtained with the self-questionnaire according to their cognitive status. AD patients were older than MCI patients and CHI (P = 0.001). There were fewer women in the group of CHI compared to MCI patients (P = 0.025) and AD patients (P = 0.004). CHI were taller than MCI patients (P = 0.043) and AD (P = 0.015). CHI and MCI patients (P = 0.017) lived more frequently at home compared to AD patients. AD patients used more frequently formal and/or informal home help services (P<0.002) compared to CHI and MCI patients. Patients with AD took more drugs than CHI (P = 0.049) and MCI patients (P = 0.047). AD patients felt more frequently discouraged and sad compared to CHI (P = 0.002). In addition, AD (P<0.001) and MCI (P = 0.008) patients had more often a positive 4-item GDS than CHI. AD patients were less independent for toileting than CHI and MCI (P = 0.043). AD patients were less independent for the abilities to use transportation independently (P<0.001) and to handle finances (P<0.002) and for the responsibility for own medications (P<0.004) compared to CHI and MCI. Patients with AD had more mobility problems than MCI (P = 0.035). Furthermore, the IADL score was lower in AD compared to CHI and MCI (P<0.002). In final, AD patients had less often a feeling happy than CHI (P = 0.002), and they practiced less often physical activity than MCI and CHI (P<0.001). There was no between-group difference for education level (10.8±3.2years for CHI, 11.5±3.2years for MCI patients, and 10.9±3.3years for AD patients, with P = 0.757). Stratification of the participants according to education level showed that there was no significant difference between self-administered questionnaire and full clinical examination among participants with higher education level (i.e., >11 years of school) and those with lower education level, exepct for number of drugs daily taken, incontinence and ADLs score (Table S3). Indeed, there was a significant difference for individuals with low level of education (i.e., ≤11 years of school) for the number of drugs daily taken (P<0.001). In addition, there was also a significant difference, whatever the education level group, for incontinence (P<0.006) and ADLs score (P<0.03).

Comparison between self- and hetero-assessments underscored that only the answers regarding height, number of drugs daily taken, ADLs score and items, and feeling of fatigue were significantly different (Table S4). AD patients declared to be taller than they actually were (P = 0.033). MCI and AD patients reported on self-administered questionnaire taking fewer drugs compared to physician assessment (P = 0.015 and P = 0.001). MCI patients declared to be less independent in ADLs (P = 0.002) and more often incontinent than estimated by the physician (P = 0.008). In contrast, CHI declared to be less often incontinent than estimated by the physician (P = 0.004). Finally, all participants declared to be more tired on self-administered questionnaire compared to physician assessment (P = 0.003), without any significant difference according to cognitive status. The magnitude of difference between results of self-questionnaire and physician examination for quantitative variables was low, regardless of the group of individuals (i.e., total population, CHI, MCI or patients with AD; please see Appendix S2).

## Discussion

The present findings show that there were few divergences between a self-administered questionnaire in older adults and a CGA performed by a physician during a full clinical examination, and that these few divergences were mainly related to the cognitive status of participants. Answers of AD and MCI patients to the self-administered questionnaire were less reliable than those of CHI.

To the best of our knowledge, we report here the first evidence that older adults are able to assess accurately their own health and functional status, with a high concordance with physician comprehensive geriatric assessment. Only few divergences were observed. First, AD patients declared to be taller than there actually were. An explanation could rely on the onset of osteoporotic vertebral fractures, which lead to decreased height in older individuals. AD patients were older than CHI and MCI patients in our study. Because of their episodic memory disorders, they were also more likely to forget and underreport this loss of height. A second divergence concerned the drugs daily taken, AD and MCI patients having declared taking fewer drugs than they actually took. This result is consistent with the fact that it may be difficult for an older adult to know precisely what kind of drugs she/he takes, particularly in the case of cognitive decline. Third, another divergence has been found with the ADLs, notably regarding incontinence among CHI and participants with MCI. An explanation could rely on the misunderstanding of the wording by participants. Indeed, incontinence is a medical term that can be unknown or misinterpreted in its definition, and thus this misunderstanding may lead to error in answers. Fourth, the ADLs score, which was calculated based on the addition of 6 items, was also divergent for patients with MCI, who answered to be more frequently dependent than they actually were. It was not the case while analyzing each item separately. Subjective perception rather than objective difficulties could be an explanation for this result. The perception of difficulties to perform activities of daily living for an individual with memory episodic disorders may be important, whilst s/he can perform them correctly, which may lead to a discrepancy between subjective perception and the absence of objective difficulties. One explanation could be related to the higher level of attention required to prepare an action compared to executing it.

Fifth, our results underscored that, regardless of their cognitive status, older patients declared to be more frequently tired on the self-administered questionnaire than they expressed to the physician. To explain this result, it could be suggested that the concept of fatigue was misinterpreted or understood differently by participants while answering to the self-administered questionnaire. It is also possible that participants showed modesty or bravery when facing the doctor, and underestimated their actual fatigue during the medical examination.

Patients' characteristics in the studied sample showed many similarities with previous studies. First, AD patients in our study were older than CHI and MCI, which is in concordance with age as a first risk factor for AD [29]–[31]. Second, participants with cognitive decline (i.e., MCI and AD) were more frequently women, fact which was also reported in previous studies [30], [31]. Third, we observed that AD patients took more drugs per day and used more frequently home services, were more depressed, practiced less physical activity and were less independent in ADLs and iADLs than CHI and MCI patients. All these characteristics are usually associated with AD and are adverse consequences of cognitive decline [30], [31]. Despite these similarities, the translation of our findings to the general elderly population should be cautious. Our sample size was relatively small and could not be calculated *a priori*. However, post-hoc power analysis using either the number of drugs for AD patients or the fact to live alone for CHI showed that the number of participants should have been between 9 and 77. Furthermore, recruitment in our study was performed in only one memory clinic. Finally, limitations include the fact that socio-economic conditions not measured could have influenced the participants' answers to the self-administered questionnaire.

The high concordance of a self-administered questionnaire and physician's CGA among older adults suggest a number of perspectives in terms of primary care. The CGA is a complex and time-consuming process that cannot be performed systematically by all general practitioners (GPs) for every geriatric patient during routine office visits [15], [16]. An active participation of the patients themselves in CGA is therefore required. We showed that a self-administered questionnaire corresponding to a self-CGA is feasible and accurate in older adults. Because the older patient may answer to the questionnaire in the waiting room, this approach is easy to implement in clinical practice. In addition, the result of this questionnaire could be used by GPs to screen frail older adults, and propose adapted care plans at the right time to the right patient.

Our results open also new perspectives in the field of QS. Nowadays, the miniaturization of devices combined with new digital technologies allow the measure of several physiological parameters reflecting health status. Therefore, QS focuses on different aspects of individuals' daily life in terms of inputs and/or outputs (e.g., food consumed, caloric expenditures, etc.), health status (e.g., blood pressure, blood oxygen levels, heart rythm, etc.), or physical performance (e.g., number of steps per day, etc.). As a result, the development of “high-tech” QS is allowing to acquire full data on older individuals, which only wait to be dissected, analyzed and interpreted. This explains that one current challenge is to transform raw data into structured and relevant material, to normalize it for analysis and to exchange it at the right time and place. As a consequence, feedbacks to individuals are still limited, and the expected beneficial impact on health remains to be demonstrated. A major forgotten step with this “high-tech” approach of QS is that individuals are not active actors of their own health, although improvements of health and functional status require individuals' active participation. Our results suggest that the use of a “low-tech” self-administered questionnaire, which captures a variety of health and functional information, could be an attractive solution for addressing this need. Among the possible perspectives, the development of a digital form accessible by touchpad or smartphone could serve both to gather health and functional information, and also to provide interactive feedback and generic and/or specific advice based on the responses entered by the patients.

In conclusion, our study shows that older adults are able to evaluate their own health and functional status, regardless of their cognitive status (i.e., CHI and patients with MCI or mild-to-moderate AD). Further research is needed to corroborate this finding but now this result opens new perspectives in the approach of geriatric patients, and could be extremely useful in daily clinical practice. A comprehensive geriatric assessment performed by an older adult could provide valuable information to the physicians to guide the medical examination and easily identify older patients requiring additional expertise and particular medical attention.

## Supporting Information

Table S1 Items of self-administered questionnaire.
(DOC)

Table S2 Participants' characteristics obtained with the self-administered questionnaire, and comparisons according to their cognitive status (n = 60). CHI = cognitively healthy individuals; MCI = mild cognitive impairment; AD =  Alzheimer disease; n: number of participants; BMI = body mass index; SD: Standard deviation; IQR: interquartile range; GDS: Geriatric depression scale; ADL: Activities of daily living; IADL: Instrumental activities of daily living; *: results from the self-administered questionnaire; †: Mild-to-moderate AD; ‡: Comparison between groups of participants based on Kruskal-Wallis with Bonferroni corrections, *t*-test, Mann-Whitney test and Chi-square test, as appropriate; §: >2 answers ‘yes’ among the 6 questions on memory complaint; #: Answer ‘happy’ or ‘very happy’ to the feeling question; ¶: Answer ‘yes’ to the question on fatigue. **: Considered if participants practiced at least one recreational physical (walking, gymnastics, cycling, swimming or gardening) activity for at least one hour a week for the past month or more; ††: A fall was defined as an event resulting in a person coming to rest unintentionally on the ground or at other lower level, not as the result of a major intrinsic event or an overwhelming hazard; P significant (<0.05) indicated in bold.
(DOC)

Table S3 P-values* of comparisons between self-administered questionnaire and physician examination according to educational level† (n = 60). n: number of participants; BMI = body mass index; SD: Standard deviation; IQR: interquartile range; ADL: Activities of daily living; IADL: Instrumental activities of daily living; ¶: Answer ‘happy’ or ‘very happy’ to the feeling question; *: Answer ‘yes’ to the question on fatigue; †: Considered if participants practiced at least one recreational physical (walking, gymnastics, cycling, swimming or gardening) activity for at least one hour a week for the past month or more; ‡: A fall was defined as an event resulting in a person coming to rest unintentionally on the ground or at other lower level, not as the result of a major intrinsic event or an overwhelming hazard; P significant (<0.05) indicated in bold.
(DOC)

Table S4 Comparisons between answers to the self-administered questionnaire and results of the full medical examination, according to the cognitive status of participants (n = 60). CHI = cognitively healthy individuals; MCI = mild cognitive impairment; AD =  Alzheimer disease; n: number of participants; BMI = body mass index; SD: Standard deviation; GDS: Geriatric depression scale; ADL: Activities of daily living; IADL: Instrumental activities of daily living; *: Mild-to-moderate AD; †: >2 answers ‘yes’ among the 6 questions on memory complaint; ‡: Item of Geriatric Depression Scale; §: Item of Activities of Daily Living scale; #: Item of Instrumental Activities of Daily Living scale; ¶: Answer ‘happy’ or ‘very happy’ to the feeling question; **: Answer ‘yes’ to the question on fatigue; ††: Considered if participants practiced at least one recreational physical (walking, gymnastics, cycling, swimming or gardening) activity for at least one hour a week for the past month or more; ‡‡: A fall was defined as an event resulting in a person coming to rest unintentionally on the ground or at other lower level, not as the result of a major intrinsic event or an overwhelming hazard; All P-value are based on paired *t*-test, Wilcoxon signed rank test or McNemar test, as appropriate.
(DOC)

Appendix S1 Items of self-administered questionnaire in French.
(DOC)

Appendix S2 Mean value, standard deviation, median and interaquartil range of difference between self-questionnaire and physician examination for quantitative variables (n = 60). CHI = cognitively healthy individuals; MCI = mild cognitive impairment; AD =  Alzheimer disease; n: number of participants; BMI = body mass index; ADLs: Activities of daily living; IADLs: Instrumental activities of daily living.
(DOC)
