# Supplementary material for: Quantified Self and Comprehensive Geriatric Assessment: Older Adults Are Able to Evaluate Their Own Health and Functional Status
Source: PLoS One. 2014 Jun 26;9(6):e100636. doi: 10.1371/journal.pone.0100636 (PMC4072604; doi:10.1371/journal.pone.0100636)
Supplement: Table S1 — Items of self-administered questionnaire. (DOC) [file pone.0100636.s001.doc]

**Table S1.** Items of self-administered questionnaire

| **Age** | 1. How old are you (years)? ¦__¦¦__¦¦__¦ |
| --- | --- |
| **Gender** | 1. Are you…? □Female or □Male |
| **Weight** | 1. Have you involuntary lost 4 kg or more in the past year? □Yes □No 2. What is your weight (in kg)? ¦__¦¦__¦¦__¦, ¦__¦ |
| **Height** | 1. What is your height (in meters)? ¦__¦,¦__¦¦__¦ |
| **Place of living** | 1. Do live at home? □Yes □No |
| **Home help** | 1. Do you receive home-help services? □Yes □No, if yes from whom? 2. □ family and/or □ friends and/or □ professionals |
| **Drugs daily taken** | 1. Number of different drugs daily taken? ¦__¦¦__¦ |
| **Memory complaint** | 1. Did someone in your environment note that you have memory   disorders? □Yes □No   1. Do you have memory disorders in activities of daily living? □Yes □No   Do you have difficulties to:   1. Calculate? □Yes □No 2. Find your words? □Yes □No 3. Learn new information? □Yes □No 4. Concentrate? □Yes □No |
| **Mood** | 1. Do you feel discouraged and sad? □Yes □No 2. Do you feel that your life is empty? □Yes □No 3. Do you feel happy most of the time? □Yes □No 4. Do you feel that your situation is hopeless? □Yes □No |
| **ADLs** | Do you need help for:   1. Toileting? □Yes □No 2. Bathing? □Yes □No 3. Dressing? □Yes □No 4. Walking and/or transferring? □Yes □No 5. Feeding? □Yes □No   Are you:   1. Incontinent? □Yes □No |
| **IADLs** | Are you able to:   1. Use the phone alone? □Yes □No 2. Use transportation alone? □Yes □No 3. Manage your own medications alone? □Yes □No 4. Handle finances alone? □Yes □No |
| **Feeling** | 1. What is your current feeling?   □Very sad, or □Sad, or □Neither one nor the other, or □Happy, or □Very happy |
| **Fatigue** | 1. Do you have a feeling of fatigue? □Yes □No   If yes: □ Exhaustion □ Physical Fatigue □ Psychological Fatigue |
| **Physical activity** | 1. Did you practice regular physical activities (walking, bicycle, etc…) at least one hour a week in the past 4 months? □Yes □No |
| **Falls** | 1. Did you fall in the previous year (at least one fall)? □Yes □No   If yes, what were the health-related adverse consequences? □ head trauma, □ fractures, □ skin damage □ inability to stand up alone, □ lying on ground > one hour |
