# Supplementary material for: Quantified Self and Comprehensive Geriatric Assessment: Older Adults Are Able to Evaluate Their Own Health and Functional Status
Source: PLoS One. 2014 Jun 26;9(6):e100636. doi: 10.1371/journal.pone.0100636 (PMC4072604; doi:10.1371/journal.pone.0100636)
Supplement: Table S2 — Participants' characteristics obtained with the self-administered questionnaire, and comparisons according to their cognitive status (n = 60). CHI = cognitively healthy individuals; MCI = mild cognitive impairment; AD = Alzheimer disease; n: number of participants; BMI = body mass index; SD: Standard deviation; IQR: interquartile range; GDS: Geriatric depression scale; ADL: Activities of daily living; IADL: Instrumental activities of daily living; *: results from the self-administered questionnaire; †: Mild-to-moderate AD; ‡: Comparison between groups of participants based on Kruskal-Wallis with Bonferroni corrections, t-test, Mann-Whitney test and Chi-square test, as appropriate; §: >2 answers ‘yes’ among the 6 questions on memory complaint; #: Answer ‘happy’ or ‘very happy’ to the feeling question; ¶: Answer ‘yes’ to the question on fatigue. **: Considered if participants practiced at least one recreational physical (walking, gymnastics, cycling, swimming or gardening) activity for at least one hour a week for the past month or more; ††: A fall was defined as an event resulting in a person coming to rest unintentionally on the ground or at other lower level, not as the result of a major intrinsic event or an overwhelming hazard; P significant (<0.05) indicated in bold. (DOC) [file pone.0100636.s002.doc]

**Table S2.** Participants' characteristics obtained with the self-administered questionnaire, and comparisons according to their cognitive status (n=60)

| Characteristics* | Total  (n=60) |  | CHI  (n=20) | MCI  (n=20) | AD†  (n=20) | P-Value‡ | | | |
| --- | --- | --- | --- | --- | --- | --- | --- | --- | --- |
|  |  |  |  |  |  | Overall | CHI  versus  MCI | CHI  versus  AD | MCI  versus  AD |
| Age (year), mean±SD and [median (IQR)] | 74.6±5.6  [73.5 (6.0)] |  | 72.3±3.0  [71.5 (5.0) ] | 72.2±2.9  [72.5 (6.0)] | 79.3±6.7  [80.0 (11.0)] | **<0.001** | 0.779 | **0.001** | **<0.001** |
| Female gender, n (%) | 31 (51.7) |  | 5 (25.0) | 12 (60) | 14 (70) | **0.011** | **0.025** | **0.004** | 0.507 |
| Involuntary weight loss >4kg in the past year, n (%) | 4 (6.7) |  | 0 (0) | 1 (5) | 3 (15) | 0.153 | 0.311 | 0.072 | 0.292 |
| Height (cm), mean±SD and [median (IQR)] | 163.2±8.6  [164.5 (13.0)] |  | 167.3±7.3  [168.5 (12.0)] | 161.9±7.3  [162.5 (12.0)] | 160.5±7.8  [160.5 (11.0)] | **0.031** | **0.043** | **0.015** | 0.478 |
| Weight (kg), mean±SD and [median (IQR)] | 68.0±12.6  [67.0 (21.0) |  | 72.3±12.4  [68.5 (21.0)] | 66.0±11.3  [66.0 (17.0)] | 65.7±13.4  [62.9 (25.0)] | 0.200 | 0.159 | 0.096 | 0.839 |
| BMI (kg/m^2^), mean±SD and [median (IQR)] | 25.4±3.9  [25.6 (5.4) |  | 25.7±3.3  [25.3 (5.3)] | 25.1±3.5  [25.7 (4.4)] | 25.4±4.8  [25.8 (7.1)] | 0.922 | 0.695 | 0.883 | 0.797 |
| Living at home, n (%) | 55 (91.7) |  | 20 (100.0) | 20 (100.0) | 15 (75.0) | **0.004** | 1.0 | **0.017** | **0.017** |
| Use of formal and/or informal home services, n (%) | 12 (20.0) |  | 0 (0) | 1 (5.0) | 11 (55.0) | **<0.001** | 0.311 | **<0.001** | **0.001** |
| Number of drugs taken daily, mean±SD and [median (IQR)] | 2.7±2.4  [2.0 (3.0)] |  | 2.5±3.1  [1.5 (3.0)] | 2.2±1.9  [2.0 (2.0)] | 3.4±1.9  [3.5 (3.0)] | 0.068 | 0.698 | **0.049** | **0.047** |
| Memory complaint§, n (%) | 38 (63.3) |  | 11 (55.0) | 11 (55.0) | 16 (80.0) | 0.166 | 1.000 | 0.691 | 0.091 |
| GDS, n (%) |  |  |  |  |  |  |  |  |  |
| Feeling discouraged and sad | 16 (26.7) |  | 1 (5.0) | 5 (25.0) | 10 (50.0) | **0.007** | 0.088 | **0.002** | 0.102 |
| Feeling that life is empty | 5 (8.3) |  | 0 (0) | 2 (10.0) | 3 (15.0) | 0.249 | 0.168 | 0.687 | 0.633 |
| Feeling happy most of the time | 55 (91.7) |  | 20 (100.0) | 17 (85.0) | 18 (90.0) | 0.217 | 0.072 | 0.147 | 0.633 |
| Feeling that situation is hopeless | 1 (1.7) |  | 0 (0) | 0 (0) | 1 (5.0) | 0.343 | 1.000 | 0.299 | 0.299 |
| 4-item GDS score >1 | 16 (26.7) |  | 0 (0) | 6 (30.0) | 10 (50.0) | **0.001** | **0.008** | **<0.001** | 0.151 |
| ADL |  |  |  |  |  |  |  |  |  |
| Toileting alone, n (%) | 57 (95.0) |  | 20 (100.0) | 20 (100.0) | 17 (85.0) | **0.043** | 1.000 | 0.072 | 0.072 |
| Bathing alone, n (%) | 58 (96.8) |  | 20 (100.0) | 20 (100.0) | 18 (90.0) | 0.126 | 1.000 | 0.147 | 0.147 |
| Dressing alone, n (%) | 58 (96.8) |  | 20 (100.0) | 20 (100.0) | 18 (90.0) | 0.126 | 1.000 | 0.147 | 0.147 |
| Walk and/or transferring alone, n (%) | 55 (91.7) |  | 20 (100.0) | 19 (95.0) | 16 (80.0) | 0.059 | 0.311 | **0.035** | 0.151 |
| Feeding alone, n (%) | 59 (98.3) |  | 20 (100.0) | 20 (100.0) | 19 (95.0) | 0.362 | 1.000 | 0.311 | 0.311 |
| Incontinence, n (%) | 34 (56.7) |  | 9 (45.0) | 12 (60.0) | 13 (65.0) | 0.414 | 0.342 | 0.204 | 0.744 |
| Total score (/6), mean±sD and [median (IQR)] | 5.3±0.8  [5.0 (1.0)] |  | 5.5±0.5  [5.0 (1.0)] | 5.5±0.5  [5.0 (1.0)] | 5.0±1.2  [5.0 (1.0)] | 0.430 | 1.000 | 0.267 | 0.267 |
| IADL |  |  |  |  |  |  |  |  |  |
| Ability to use the phone, n (%) | 59 (98.3) |  | 20 (100.0) | 20 (100.0) | 19 (95.0) | 0.362 | 1.000 | 0.311 | 0.311 |
| Ability to use transportation independently, n (%) | 45 (75.0) |  | 20 (100.0) | 18 (90.0) | 7 (35.0) | **<0.001** | 0.147 | **<0.001** | **<0.001** |
| Responsibility for own medications, n (%) | 50 (83.3) |  | 20 (100.0) | 19 (95.0) | 11 (55.0) | **<0.001** | 0.311 | **0.001** | **0.003** |
| Ability to handle finances, n (%) | 45 (75.0) |  | 20 (100.0) | 18 (90.0) | 8 (40.0) | **<0.001** | 0.548 | **<0.001** | **0.001** |
| Total score (/4), mean±SD and [median (IQR)] | 3.3±1.2  [4.0 (1.0)] |  | 4.0±0.2  [4.0 (0)] | 3.7±0.8  [4.0 (0)] | 2.3±1.4  [2.0 (3.0)] | **<0.001** | 0.150 | **<0.001** | **0.001** |
| Feeling happy to very happy#, n(%) | 40 (66.7) |  | 18 (90.0) | 13 (65.0) | 9 (45.0) | **0.010** | 0.058 | **0.002** | 0.204 |
| Fatigue¶, n(%) | 42 (70.0) |  | 13 (65.0) | 13 (65.0) | 16 (80.0) | 0.490 | 1.000 | 0.288 | 0.288 |
| Practice physical activity**, n(%) | 47 (78.3) |  | 20 (100.0) | 19 (95.0) | 8 (40.0) | **<0.001** | 0.311 | **<0.001** | **<0.001** |
| History of falls in past years ††, n (%) | 14 (23.3) |  | 2 (10.0) | 5 (25.0) | 7 (35.0) | 0.170 | 0.212 | 0.058 | 0.490 |

CHI=cognitively healthy individuals

MCI=mild cognitive impairment

AD= Alzheimer disease

n: number of participants

BMI=body mass index

SD: Standard deviation

IQR: interquartile range

GDS: Geriatric depression scale

ADL: Activities of daily living

IADL: Instrumental activities of daily living

*: results from the self-administered questionnaire

†: Mild-to-moderate AD

‡: Comparison between groups of participants based on Kruskal-Wallis with Bonferroni corrections, *t*-test, Mann-Whitney test and Chi-square test, as appropriate

§: >2 answers 'yes' among the 6 questions on memory complaint

#: Answer 'happy' or 'very happy' to the feeling question

¶: Answer 'yes' to the question on fatigue

**: Considered if participants practiced at least one recreational physical (walking, gymnastics, cycling, swimming or gardening) activity for at least one hour a week for the past month or more.

††: A fall was defined as an event resulting in a person coming to rest unintentionally on the ground or at other lower level, not as the result of a major intrinsic event or an overwhelming hazard.

P significant (<0.05) indicated in bold.
