# Supplementary material for: Quantified Self and Comprehensive Geriatric Assessment: Older Adults Are Able to Evaluate Their Own Health and Functional Status
Source: PLoS One. 2014 Jun 26;9(6):e100636. doi: 10.1371/journal.pone.0100636 (PMC4072604; doi:10.1371/journal.pone.0100636)
Supplement: Table S3 — P-values* of comparisons between self-administered questionnaire and physician examination according to educational level† (n = 60). n: number of participants; BMI = body mass index; SD: Standard deviation; IQR: interquartile range; ADL: Activities of daily living; IADL: Instrumental activities of daily living; ¶: Answer ‘happy’ or ‘very happy’ to the feeling question; *: Answer ‘yes’ to the question on fatigue; †: Considered if participants practiced at least one recreational physical (walking, gymnastics, cycling, swimming or gardening) activity for at least one hour a week for the past month or more; ‡: A fall was defined as an event resulting in a person coming to rest unintentionally on the ground or at other lower level, not as the result of a major intrinsic event or an overwhelming hazard; P significant (<0.05) indicated in bold. (DOC) [file pone.0100636.s003.doc]

**Table S3.** P-values* of comparisons between self-administered questionnaire and physician examination according to educational level† (n=60)

| Characteristics | Number of years of school† | |
| --- | --- | --- |
|  | < 11  (n=20) | >11  (n=20) |
| Age | 0.140 | 0.655 |
| Female gender | 0.648 | 0.753 |
| Involuntary weight loss >4kg in past year | 0.556 | 0.549 |
| Height (cm) | 0.111 | 0.705 |
| Weight (kg) | 0.081 | 0.194 |
| BMI (kg/m^2^) | 0.958 | 1.000 |
| Living at home | 0.723 | 0.293 |
| Use of formal and/or informal home services | 0.300 | 0.707 |
| Number of drugs taken daily | **<0.001** | 0.054 |
| Memory complaint§ | 1.000 | 0.212 |
| GDS |  |  |
| Feeling discouraged and sad | 0.444 | 0.939 |
| Feeling that life is empty | 0.249 | 0.269 |
| Feeling happy most of the time | 0.455 | 1.000 |
| Feeling that situation is hopeless | 0.556 | 0.323 |
| 4-item GDS score >1 | 1.000 | 0.731 |
| ADL |  |  |
| Toileting alone | 0.288 | 1.000 |
| Bathing alone, | 0.644 | 1.000 |
| Dressing alone, | 0.556 | 0.972 |
| Walk and/or transferring alone, | 0.644 | 0.948 |
| Feeding alone | 1.000 | 1.000 |
| Incontinence | **0.002** | **0.005** |
| Total score (/6) | **0.025** | **0.003** |
| IADL |  |  |
| Ability to use the phone | 0.165 | 1.000 |
| Ability to use transportation independently | 0.624 | 0.959 |
| Responsibility for own medications | 0.508 | 0.959 |
| Ability to handle finances | 0.804 | 0.731 |
| Total score (/4) | 0.223 | 1.000 |
| Feeling happy to very happy# | 1.000 | 0.753 |
| Fatigue¶ | 0.111 | 0.172 |
| Practice physical activity* | 0.792 | 0.469 |
| History of falls in past year † | 0.429 | 1.000 |

n: number of participants

BMI=body mass index

SD: Standard deviation

IQR: interquartile range

ADL: Activities of daily living

IADL: Instrumental activities of daily living

¶: Answer 'happy' or 'very happy' to the feeling question

*: Answer 'yes' to the question on fatigue

†: Considered if participants practiced at least one recreational physical (walking, gymnastics, cycling, swimming or gardening) activity for at least one hour a week for the past month or more.

‡: A fall was defined as an event resulting in a person coming to rest unintentionally on the ground or at other lower level, not as the result of a major intrinsic event or an overwhelming hazard.

P significant (<0.05) indicated in bold.
