# Supplementary material for: Quantified Self and Comprehensive Geriatric Assessment: Older Adults Are Able to Evaluate Their Own Health and Functional Status
Source: PLoS One. 2014 Jun 26;9(6):e100636. doi: 10.1371/journal.pone.0100636 (PMC4072604; doi:10.1371/journal.pone.0100636)
Supplement: Table S4 — Comparisons between answers to the self-administered questionnaire and results of the full medical examination, according to the cognitive status of participants (n = 60). CHI = cognitively healthy individuals; MCI = mild cognitive impairment; AD = Alzheimer disease; n: number of participants; BMI = body mass index; SD: Standard deviation; GDS: Geriatric depression scale; ADL: Activities of daily living; IADL: Instrumental activities of daily living; *: Mild-to-moderate AD; †: >2 answers ‘yes’ among the 6 questions on memory complaint; ‡: Item of Geriatric Depression Scale; §: Item of Activities of Daily Living scale; #: Item of Instrumental Activities of Daily Living scale; ¶: Answer ‘happy’ or ‘very happy’ to the feeling question; **: Answer ‘yes’ to the question on fatigue; ††: Considered if participants practiced at least one recreational physical (walking, gymnastics, cycling, swimming or gardening) activity for at least one hour a week for the past month or more; ‡‡: A fall was defined as an event resulting in a person coming to rest unintentionally on the ground or at other lower level, not as the result of a major intrinsic event or an overwhelming hazard; All P-value are based on paired t-test, Wilcoxon signed rank test or McNemar test, as appropriate. (DOC) [file pone.0100636.s004.doc]

**Table S4.** Comparisons between answers to the self-administered questionnaire and results of the full medical examination, according to the cognitive status of participants (n=60)

|  | Total  (n=60) |  | CHI  (n=20) | MCI  (n=20) | AD*  (n=20) |
| --- | --- | --- | --- | --- | --- |
| Comparisons of quantitative variables |  |  |  |  |  |
| Age | 0.291 |  | 0.157 | 0.285 | 0.109 |
| Height | 0.125 |  | 0.739 | 0.180 | 0.033 |
| Weight | 0.269 |  | 0.414 | 0.414 | 0.152 |
| BMI | 0.900 |  | 0.878 | 0.225 | 0.600 |
| Number of drugs taken daily | <0.001 |  | 0.483 | 0.015 | 0.001 |
| ADLs score (/6) | <0.001 |  | 0.052 | 0.002 | 0.414 |
| IADLs score (/4) | 0.276 |  | 0.414 | 0.157 | 0.222 |

Comparisons of qualitative variables

| Female gender, | 0.375 |  | 1.000 | 1.000 | 0.625 | |
| --- | --- | --- | --- | --- | --- | --- |
| Involuntary weight loss >4kg in the past year | 0.500 |  | 1.000 | 1.000 | 0.500 | |
| Living at home | 0.250 |  | 0.500 | 1.000 | 1.000 | |
| Use of formal and/or informal home services | 0.063 |  | 1.000 | 1.000 | 0.250 | |
| Memory complaint† | 0.454 |  | 0.125 | 1.000 | 0.688 | |
| Feeling discouraged and sad‡ | 0.250 |  | 1.000 | 1.000 | 0.250 | |
| Feeling that life is empty‡ | 1.000 |  | 1.000 | 0.500 | 0.500 | |
| Feeling happy most of the time‡ | 0.727 |  | 1.000 | 1.000 | 0.625 | |
| Feeling that situation is hopeless‡ | 0.625 |  | 1.000 | 1.000 | 1.000 | |
| 4-item GDS score >1 | 1.000 |  | 0.500 | 1.000 | 1.000 | |
| Toileting§ | 0.250 |  | 1.000 | 1.000 | 0.500 | |
| Bathing§ | 1.000 |  | 1.000 | 1.000 | 1.000 | |
| Dressing§ | 1.000 |  | 1.000 | 1.000 | 1.000 | |
| Walking and/or transferring§ | 1.000 |  | 1.000 | 1.000 | 1.000 | |
| Feeding§ | 1.000 |  | 1.000 | 1.000 | 1.000 | |
| Incontinence§ | <0.001 |  | 0.004 | 0.008 | 0.250 | |
| Ability to use the phone# | 0.375 |  | 1.000 | 1.000 | 0.625 | |
| Ability to use transportation independently# | 0.500 |  | 1.000 | 1.000 | 1.000 | |
| Responsibility for own medications # | 0.375 |  | 1.000 | 1.000 | 0.625 | |
| Ability to handle finances# | 1.000 |  | 1.000 | 1.000 | 1.000 | |
| Feeling happy to very happy¶ | 1.000 |  | 1.000 | 1.000 | | 0.625 |
| Fatigue** | 0.003 |  | 0.063 | 0.125 | | 0.625 |
| Practice physical activity†† | 0.250 |  | 1.000 | 1.000 | | 1.000 |
| History of falls in past years ‡‡ | 0.250 |  | 1.000 | 1.000 | | 0.500 |

CHI=cognitively healthy individuals

MCI=mild cognitive impairment

AD= Alzheimer disease

n: number of participants

BMI=body mass index

SD: Standard deviation

GDS: Geriatric depression scale

ADL: Activities of daily living

IADL: Instrumental activities of daily living

*: Mild-to-moderate AD

†: >2 answers 'yes' among the 6 questions on memory complaint

‡: Item of Geriatric Depression Scale

§: Item of Activities of Daily Living scale

#: Item of Instrumental Activities of Daily Living scale

¶: Answer 'happy' or 'very happy' to the feeling question

**: Answer 'yes' to the question on fatigue

††: Considered if participants practiced at least one recreational physical (walking, gymnastics, cycling, swimming or gardening) activity for at least one hour a week for the past month or more.

‡‡: A fall was defined as an event resulting in a person coming to rest unintentionally on the ground or at other lower level, not as the result of a major intrinsic event or an overwhelming hazard.

All P-value are based on paired *t*-test, Wilcoxon signed rank test or McNemar test, as appropriate.

P-values indicated in different colors, as follows:

|  | >0.600 |
| --- | --- |
|  | 0.599-0.400 |
|  | 0.399-0.201 |
|  | 0.200-0.05 |
|  | <0.05 |
