# Supplementary material for: Quantified Self and Comprehensive Geriatric Assessment: Older Adults Are Able to Evaluate Their Own Health and Functional Status
Source: PLoS One. 2014 Jun 26;9(6):e100636. doi: 10.1371/journal.pone.0100636 (PMC4072604; doi:10.1371/journal.pone.0100636)
Supplement: Appendix S1 — Items of self-administered questionnaire in French. (DOC) [file pone.0100636.s005.doc]

**Appendix S1.** Items of self-administered questionnaire in French

| **Age (en années)** | 1. Quel est votre age ? ¦__¦¦__¦ |
| --- | --- |
| **Sexe** | 2. Etes-vous une □ Femme ou un □ Homme ? |
| **Poids** | 3. Avez-vous perdu 4 kg involontairement au cours de la dernière année ? □ Oui □ Non  4. Quel est votre poids (en kg) ? ¦__¦¦__¦¦__¦, ¦__¦ |
| **Taille** | 5. Quel est votre taille (en mètres) ? ¦__¦,¦__¦¦__¦ |
| **Lieu de vie** | 6. Vivez–vous à Domicile ? □ Oui □ Non |
| **Aides dans la vie de tous les jours à domicile** | 7. Recevez-vous de l'aide à domicile ? □ Oui □ Non,  8. Si oui de qui : d'un □ parent et/ou □ ami et/ou □ professionnel |
| **Prise médicaments** | 9. Combien de médicaments différents prenez-vous par jour ? ¦__¦¦__¦ |
| **Plainte mémoire** | 10. Votre entourage vous a-t-il fait remarquer que vous aviez des troubles de la mémoire ? □ Oui □Non  11. Avez-vous des trous de mémoire dans la vie de tous les jours ? □ Oui □ Non  Avez-vous des difficultés pour :  12. Calculer ? □ Oui □ Non  13. Trouvez vos mots ? □ Oui □ Non  14. Mémoriser de nouvelles informations ? □ Oui □ Non  15. Vous concentrer ? □ Oui □ Non |
| **Etat d'humeur** | 16. Vous sentez-vous souvent découragé(e) et triste ? □ Oui □ Non  17. Avez-vous le sentiment que votre vie est vide ? □ Oui □ Non  18. Etes-vous heureux (se) la plupart du temps ? □ Oui □ Non  19. Avez-vous l'impression que votre situation est désespérée ? □ Oui □ Non |
| **Actes de la vie quotidienne liés à vos capacités motrices** | Avez-vous besoin d'aide pour :  20. Faire votre toilette ? □ Oui □ Non  21. Faire vos soins d'apparence corporelle ? □ Oui □ Non  22. Vous habiller ? □ Oui □ Non  23. Vous lever et/ou vous déplacer ? □ Oui □ Non  24. Manger ? □ Oui □ Non  25. Etes-vous incontinent ? □ Oui □ Non |
| **Actes de la vie quotidienne liés à vos capacités intellectuelles** | Êtes-vous capable :  26. Utiliser seul le téléphone ? □ Oui □ Non  27. Utiliser seul les moyens de transport ? □ Oui □ Non  28. De prendre seul vos médicaments ? □ Oui □ Non  29. De gérer seul votre budget ? □ Oui □ Non |
| **Sentiment général** | 30. Quel est votre sentiment actuel? □ Très malheureux □ Malheureux □ Ni l'un ni l'autre □ Heureux □ Très heureux |
| **Etat de Fatigue** | 31. Vous sentez-vous fatigué ? □ Oui □ Non  si oui : □ Epuisement □ Fatigue physique □ Fatigue psychique |
| **Activité physique** | 32. Avez-vous pratiqué une activité physique (marche, jardinage, vélo, etc…) au moins pendant 1 heure par semaine au cours du dernier mois? □ Oui □ Non |
| **Chute** | 33. Avez-vous au cours de l'année précédente :  - Au moins fait une chute ? □ Oui □ Non  - Si oui, la ou les chutes se sont-elles compliquées □ d'un traumatisme crânien, □ de fractures, □ d'une plaie cutanée avec suture, □ l'impossibilité de se relever seul du sol,  □ d'une durée de séjour sur le sol de plus d'une heure ? |
