# Supplementary material for: Quantified Self and Comprehensive Geriatric Assessment: Older Adults Are Able to Evaluate Their Own Health and Functional Status
Source: PLoS One. 2014 Jun 26;9(6):e100636. doi: 10.1371/journal.pone.0100636 (PMC4072604; doi:10.1371/journal.pone.0100636)
Supplement: Appendix S2 — Mean value, standard deviation, median and interaquartil range of difference between self-questionnaire and physician examination for quantitative variables (n = 60). CHI = cognitively healthy individuals; MCI = mild cognitive impairment; AD = Alzheimer disease; n: number of participants; BMI = body mass index; ADLs: Activities of daily living; IADLs: Instrumental activities of daily living. (DOC) [file pone.0100636.s006.doc]

**Appendix S2.** Mean value, standard deviation, median and interaquartil range of difference between self-questionnaire and physician examination for quantitative variables (n=60)

| Difference between self-questionnaire and physician examination, mean±SD and [median (IQR)] | Total  (n=60) |  | CHI  (n=20) | MCI  (n=20) | AD  (n=20) |
| --- | --- | --- | --- | --- | --- |
| Age (year) | 0.29±1.72  [0.00 (0.00)] |  | 0.11±0.32  [0.00 (0.00)] | -0.20±0.83  [0.00 (0.00)] | 0.95±2.74  [0.00 (0.00)] |
| Height (cm) | 0.81±4.87  [0.00 (0.00)] |  | 0.05±0.70 [0.00 (0.00)] | 0.70±2.09  [0.00 (0.00)] | 3.05±5.86  [0.00 (5.25)] |
| Weight (kg) | 0.60±2.86  [0.00 (0.00)] |  | -0.11±0.57  [0.00 (0.00)] | 0.10±0.55  [0.00 (0.00)] | 1.77±4.7  [0.00 (2.75)] |
| BMI (kg/m^2^) | -0.03±1.53  [0.00 (0.30)] |  | 0.05±0.320 [0.00 (0.35)] | 0.27±1.00  [0.00 (0.00)] | -0.32±2.41  [0.00 (0.70)] |
| Number of drugs taken daily | -1.49±2.34  [0.00 (2.00)] |  | -0.26±0.65  [0.00 (1.00)] | -1.15±2.03  [-0.50 (2.00)] | -3.00±2.85  [-2.50 (5.00)] |
| ADLs score (/6) | -0.25±0.88  [0.00 (1.00)] |  | -0.16±1.34  [0.00 (1.00)] | -0.50±0.51  [-0.50 (1.00)] | -0.10±0.55  [0.00 (0.00)] |
| IADLs score (/4) | 0.12±0.83  [0.00 (0.00)] |  | 0.16±0.96  [0.00 (0.00)] | -0.10±0.31  [0.00 (0.00)] | 0.30±1.03  [0.00 (1.00)] |

CHI=cognitively healthy individuals; MCI=mild cognitive impairment; AD= Alzheimer disease; n: number of participants; BMI=body mass index; ADLs: Activities of daily living

IADLs: Instrumental activities of daily living
